# Viroplasms: Assembly and Functions of Rotavirus Replication Factories

**DOI:** 10.3390/v13071349

**Published:** 2021-07-12

**Authors:** Guido Papa, Alexander Borodavka, Ulrich Desselberger

**Affiliations:** 1 MRC Laboratory of Molecular Biology, Cambridge Biomedical Campus, Cambridge CB2 0QH, UK; gpapa@mrc-lmb.cam.ac.uk; 2 Department of Biochemistry, University of Cambridge, Cambridge CB2 1QW, UK; ab2677@cam.ac.uk; 3 Department of Medicine, Addenbrooke’s Hospital, University of Cambridge, Cambridge CB2 0QQ, UK

**Keywords:** rotavirus, viroplasm, replication cycle, NSP5, NSP2, reverse genetics, CRISPR-Csy4 genome editing, protein-RNA condensates, liquid-liquid phase separation

## Abstract

Viroplasms are cytoplasmic, membraneless structures assembled in rotavirus (RV)-infected cells, which are intricately involved in viral replication. Two virus-encoded, non-structural proteins, NSP2 and NSP5, are the main drivers of viroplasm formation. The structures (as far as is known) and functions of these proteins are described. Recent studies using plasmid-only-based reverse genetics have significantly contributed to elucidation of the crucial roles of these proteins in RV replication. Thus, it has been recognized that viroplasms resemble liquid-like protein–RNA condensates that may be formed via liquid–liquid phase separation (LLPS) of NSP2 and NSP5 at the early stages of infection. Interactions between the RNA chaperone NSP2 and the multivalent, intrinsically disordered protein NSP5 result in their condensation (protein droplet formation), which plays a central role in viroplasm assembly. These droplets may provide a unique molecular environment for the establishment of inter-molecular contacts between the RV (+)ssRNA transcripts, followed by their assortment and equimolar packaging. Future efforts to improve our understanding of RV replication and genome assortment in viroplasms should focus on their complex molecular composition, which changes dynamically throughout the RV replication cycle, to support distinct stages of virion assembly.

## 1. Introduction

Species A rotaviruses (RVs) are a major cause of acute gastroenteritis in infants and young children worldwide [1]. Despite the effectiveness and availability of several RVvaccines [2], RV-associated deaths in children < 5 years of age are still a problem, particularly in countries in sub-Saharan Africa and SE Asia [3]. Despite extensive investigations that haven taken place over 50 years, and the significant progress made during the last decade, there are still aspects of the molecular biology, pathogenesis and immunology of these viruses that are poorly understood. This review will concentrate on cytoplasmic sites of RV replication, or viroplasms, also known as RV replication factories.

## 2. Rotavirus Particle Structure and Replication Cycle

**Figure 1:**
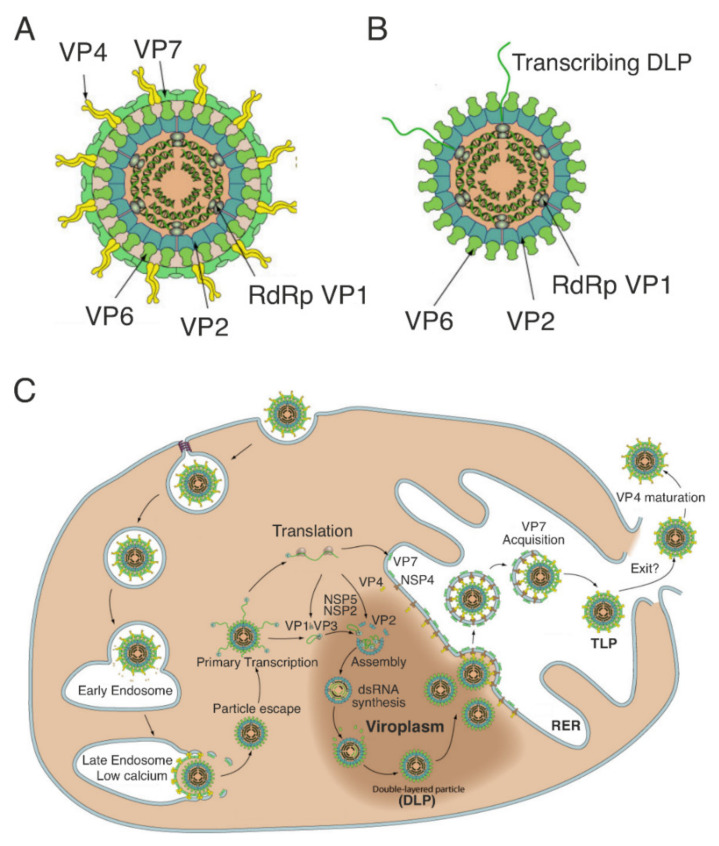
Rotavirus structure, replication cycle. (**A**,**B**) Schematic representation of triple-layered and double-layered rotavirus particles (TLPs and DLPs, respectively). Outer layer: VP7, VP4 (spikes); middle layer: VP6; inner layer (core): VP2. The core contains 11 segments of dsRNA (diagrammatically shown as concentric rings) and the viral transcription–replication complex, consisting of the RNA-dependent RNA polymerase (RdRp), VP1, and the capping enzyme, VP3. Rotavirus DLPs are transcription active, producing (+)ssRNAs from each genomic RNA segment. Adapted from: ViralZone, SIB Swiss Institute of Bioinformatics; (**C**) Rotavirus replication cycle (diagram) including formation of viroplasms. For details, see text. Adapted from: ViralZone, SIB Swiss Institute of Bioinformatics.

Rotavirus is a non-enveloped RNA virus whose genome is composed of 11 segments of double-stranded (ds) RNA encoding six structural proteins (VP1-VP4, VP6, VP7) and 5-6 non-structural proteins (NSP1-NSP5/NSP6). The virion particle is composed of three protein layers and is also known as a Triple-Layered Particle (TLP). During the initial phases of the replication cycle, the virus loses the external layer, consisting of VP7 and VP4, giving rise to a transcriptionally active subviral particle, called a Double-Layered Particle (DLP). The outside of the DLP is the intermediate layer, consisting of 260 VP6 trimers, and encloses the core of 120 molecules of VP2, arranged as 60 dimers. The core contains the genomic dsRNA segments, the RNA-dependent RNA polymerase (RdRp), VP1, and the capping enzyme, VP3. The DLP produces capped, non-polyadenylated (+)ssRNAs, which are released into the cytoplasm through the channels located at the five-fold symmetry axes of the particle (Figure 1A) [1,4].

The RV replication cycle (Figure 1B) begins with TLP’s interaction with the host attachment receptors (sialoglycans and histo-blood group antigens) via the VP8* subunit of the outer capsid protein VP4 [5,6]. Following endocytosis, the viral particle is engulfed into endosomes, where Ca^2+^ levels are low: this induces the loss of theVP4–VP7 outer layer and the release of a transcriptionally active DLP into the cytosol. Capped (+)ssRNAs are transcribed from each of the 11 dsRNA genome segments. Using fluorescently tagged outer-layer proteins and fluorescent in situ hybridization probes for nascent transcripts, uncoating of and transcription by RV particles were shown to occur as early as 15 min after virus adsorption [7]. The (+)ssRNAs are either translated, or serve as templates for the synthesis of negative-sense RNA ((–)RNA) during replication of the viral genome. The interaction between the virus-encoded Non-Structural Protein 2 (NSP2) and Non-Structural Protein 5 (NSP5) results in the formation of 0.1–5 μm cytoplasmic inclusions, termed viroplasms, which appear to concentrate the viral components required for genome assortment and replication, and the assembly of subviral particles, thus acting as ‘viral factories’. Within these NSP5/NSP2-rich inclusions, interactions between several viral proteins (VP1, VP3, VP2, VP6, and NSP2) with the pre-genomic (+)ssRNA, result in their stoichiometric co-packaging into early assembly intermediates. Inside these, VP1/VP2 interactions trigger dsRNA synthesis [8]. The intermediate capsid protein, VP6, is believed to assemble onto the nascent core to form the DLPs, which bud from viroplasms into adjacent cellular membranes, where NSP4 is inserted, acting as an intracellular receptor for DLPs [9]. DLPs then acquire the membrane-resident outer-capsid proteins, VP7, and the spike protein, VP4, forming a temporary enveloped particle; the envelope is lost during the formation of the infectious TLP [1,10]. Our understanding of the key steps of RV virion assembly is limited, and the exact timing of these events and their spatio-temporal control remain to be explored.

## 3. Viroplasm Formation and Functions of NSP5 and NSP2

**Figure 2:**
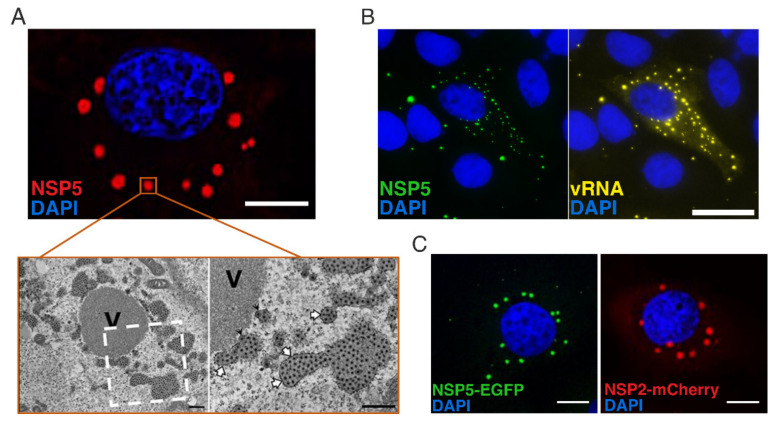
Rotavirus viroplasm formation. (**A**) Formation of rotavirus viroplasm. Upper panel: Immunofluorescent image of a rotavirus-infected cell at 10 hpi, stained with anti-NSP5 antibody (red) and DAPI (blue). Scale bar, 10 μm. Lower panel: Electron micrographs of MA104 cells infected with wildtype (WT) rotavirus (MOI, 75 FFU/cell) at 10 hpi. V, viroplasm. The white open box denotes an area shown at higher magnification in the right-hand EM photograph. White arrows indicate endoplasmic membranes surrounding viroplasms; black arrowheads indicate viral particles with an envelope. Scale bar, 500 nm. Adapted with permission from Papa et al. [25]; (**B**) Confocal imaging of rotavirus transcripts via fluorescence in situ hybridisation (FISH). FISH probes specific to the rotavirus transcripts (segment 1–11) were used to visualise RV transcripts in EGFP-NSP5-tagged viroplasms (green). RNA signal is shown in yellow (Quasar 560-labelled probes). Nuclei were stained with DAPI (blue), Scale bar, 10 μm. Adapted from Strauss et al. [26]; (**C**) Rotavirus infection of cells constitutively expressing fluorescently labelled NSP2 and NSP5 proteins. Recruitment of NSP5-EGFP in green (left) or NSP2-mCherry in red (right) proteins to viroplasms in rotavirus-infected MA104 cells constitutively expressing the two fluorescent fusion proteins. DAPI (blue) detects nuclei. Scale bar: 10 μm. From Papa [27].

Viroplasms can be microscopically detected as early as 2 h post-infection (h.p.i.) onwards by using anti-NSP5 or NSP2-specific antibodies [11]. By electron and fluorescent microscopy, viroplasms are membraneless inclusions, where viral mRNAs are accumulated, packaged and replicated to form new DLPs, which are released into the cytosol (Figure 2A,B). While the complete structure of NSP5 remains unknown, NSP2 forms ~300 kDa large octamers that possess an RNA-helix destabilizing, nucleoside diphosphate kinase [12,13,14], and RNA chaperone activities [15,16]. In contrast, the 22 kDa serine/threonine-rich acidic protein NSP5 is believed to assemble into higher-order species (dimers, decamers and larger oligomers of poorly defined stoichiometry) in solution [17], has an ATPase activity [18] and can undergo phosphorylation of its several serine residues, i.e., become hyperphosphorylated [19,20]. NSP2-NSP5 interactions are crucial in the formation of viroplasms, as the co-expression of these two proteins in uninfected cells leads to the formation of viroplasm-like structures (VLSs) [21]. Furthermore, the inhibition of either NSP2 or NSP5 expression in RV-infected cells prevents viroplasm formation and drastically impacts the production of infectious viral progeny, further proving their pivotal role in assembling viroplasms [22,23,24,25].

By applying and modifying one of the recently developed, plasmid-only-based reverse genetics systems for RVs [28,29], Papa et al. [25] provided direct evidence of the crucial role of NSP5 in rotavirus replication by generating a recombinant RV (rRV) unable to produce NSP5. The NSP5-deficient rRV could only be rescued and replicated in MA104 cells expressing the wildtype (wt) NSP5 gene in *trans*, thus directly demonstrating the essential function of NSP5 for rotavirus replication (Figure 2C).

**Figure 3:**
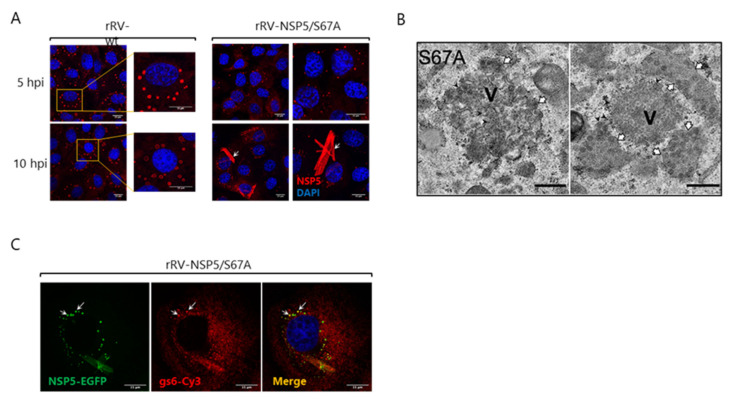
Replication of rRV-NSP5/S67A mutant: Pivotal role of phosphorylation in assembling round-shaped and fully functional viroplasms. (**A**) Immunofluorescent images of MA104 cells infected with rRV-wt and rRV-NSP5/S67A (MOI of 15 FFU/cell) at 5 hpi (upper panels) or 10 hpi (lower panels). Cells were stained with anti-NSP5 (red) and DAPI (blue). Scale bar, 15 μm. From Papa et al. [25]; (**B**) Electron microscopy image of MA104 cells infected with rRV-NSP5/S67A (MOI, 75 FFU/cell) at 10 hpi. V, viroplasm. White arrows indicate the endoplasmic reticulum surrounding viroplasms; black arrowheads indicate putative viral particles with an envelope. Scale bar, 500 nm. From Papa et al. [25]; (**C**) RNA Fluorescence In Situ Hybridisation (RNA-FISH) on MA104-NSP5-EGFP cells infected with rRV-NSP5/S67A. Viroplasms detected with NSP5-EGFP (green) and viral RNA (probe specific for genome segment 6-specific probe was with Cy3 (red). Co-localization of viroplasms and RNA is indicated by white arrows. Scale bar, 15 μm. From Papa et al. [25].

This system also enabled the rescue of several rRV mutants with modifications in the NSP5-encoding genomic segment, including an rRV with impaired NSP5 phosphorylation due to a Ser67 to Ala substitution (S67A). rRV-NSP5/S67A produced an unphosphorylated NSP5 and assembled aberrant spindle-like viroplasms (Figure 3A), which were impaired in supporting viral replication. Compared to RV wt-infected cells, the transmission EM of rRV-NSP5/S67A-infected cells showed much lower numbers of RV particles (compare Figure 3B and Figure 2A) and a lower content of viral RNA (compare Figure 3C with Figure 2B), thus leading to a strong reduction (~100-fold) in virus yield [25]. These data demonstrated that the hyperphosphorylation of NSP5 is important for the assembly of fully functional RV viral factories.

Using the same trans-complementing approach, other NSP5 mutants harbouring deletions in NSP5 C-terminal region were rescued. These viruses produced an unphosphorylated NSP5 and were completely unable to form viroplasms and replicate, demonstrating the pivotal role of the C-terminal region of NSP5 in hyperphosphorylation and viroplasm assembly [25].

While the exact role of phosphorylation of NSP5 remains poorly understood, the results suggest that phosphorylation may control the correct assembly of viroplasms by modulating the interactions between NSP5 and NSP2. Silencing of the cellular phosphorylating enzyme CK1α prevented NSP2 phosphorylation on serine 313 [30]. Using an RG approach, this group has also demonstrated the role of NSP2 phosphorylation during rotavirus infection by producing an rRV containing a phosphomimetic NSP2 (mutant S313D). Similar to the S67A NSP5 mutant, which showed impaired replication in MA104 cells, the S313D NSP2 mutant remained replication-competent, but showed a delay in viroplasm formation and a decrease in virus progeny production [31,32]. Together, these novel results, obtained using RG approaches, reveal the complex roles of viroplasmic protein phosphorylation in RV replication.

Since they are two major protein components of viroplasms, NSP5 and NSP2 have been exploited as “shuttle proteins” to localise fluorescent proteins to viroplasms. This approach has been applied to generate stable cell lines expressing NSP5 fused to Enhanced Green Fluorescent Protein (EGFP), or NSP2 fused to the mCherry protein, allowing for the investigation of the dynamics of viroplasm formation (Figure 2C) [19,25].

Similarly, other non-viral proteins, e.g., CRISPR-associated endonucleases, have been successfully fused to NSP5, permitting the endonucleolytic cleavage of recombinant viral RNAs inside viroplasms [33]. These experiments have directly confirmed that viroplasms represent the sites of RV genome replication, since localisation of the Csy4 endonuclease to viroplasms resulted in the editing of specific genomic segments. Moreover, this approach has also allowed for modification of the transcripts produced during the ‘secondary transcription’ by newly assembled DLPs, demonstrating that this step largely contributes to the overall production of viral proteins during infection [33], thus providing a molecular mechanism of earlier observations [34].

## 4. Viroplasms Are Protein-RNA Condensates

Recently, viroplasms were recognized as protein-RNA condensates formed by phase separation of the RV non-structural proteins NSP2 and NSP5. At early stages of infection, the condensates could be reversibly dissolved by small aliphatic diols, such as 1,6-hexanediol, 1,5-pentanediol, 1,4-butanediol and 1.2- and 1.3-propylene diols. At late stages of infection, larger, less spherical viroplasms [11] were insensitive to aliphatic diols, suggesting a transition from a liquid to a gel- or solid-like state [35]. Likewise, similar liquid-like properties were reported for the replication factories of other RNA viruses, including rabies virus [36,37,38], vesicular stomatitis virus [39], influenza virus [40], respiratory syncytial virus [41], measles virus [42,43], and SARS-CoV-2 [44,45,46].

The significance of liquid–liquid phase separation (LLPS) in the molecular biology of these viruses remains to be further explored. Similar LLPS-driven phenomena are increasingly being reported in diverse other biological systems [47,48,49,50,51], where biomolecular condensates may be involved in RNA metabolism, DNA damage response, and signal transduction [48]. The major challenges in understanding the role of LLPS in viral replication include the limited knowledge of major determinants of phase separation. Detailed quantitative characterisation of the phase behaviour of these protein-RNA condensates would permit the introduction of specific mutations that abrogate LLPS or shift the phase boundary of the condensate, as well as the study of the role of LLPS in the context of RV replication. The possible implications of RV replication within such condensates will be discussed below.

## 5. Interaction of Viroplasms with Cellular Components

Apart from the main components NSP2 and NSP5, viroplasms also contain other viral components, including (+)ssRNAs of all 11 genome segments, VP1, VP3, VP2 and VP6, which are key players in the RV replication and production of new viral progeny particles during infection [52].

Despite their high selectivity, unsurprisingly, viroplasms also interact with numerous cellular components, including lipids, proteins and miRNAs. At 5-6 h p.i., viroplasms start to form complexes with the cellular organelles lipid droplets (LDs) [52,53,54,55]. LDs are a storage compartment for energy sources (triacylglycerols, sterol esters), surrounded by phospholipid monolayers, into which >100 cellular proteins are inserted [56]. In addition, LDs are dynamic organelles involved in signal transduction and membrane trafficking [57,58,59]. The inhibition of LD formation (biogenesis) or the stimulation of their lysis (lipolysis) by chemical compounds at non-toxic concentrations were shown to decrease the number and size of viroplasms and the yield of infectious viral progeny [52,55,60]. LDs also interact with VLSs [52], emphasizing their role in the interaction with NSP2 and NSP5 in viroplasm formation. Cellular proteins interacting with LDs often contain amphipathic helices [61], as does the rotavirus NSP5 [62]. However, details of the molecular mechanisms involved in viroplasm-LD interactions remain to be explored. In this context, it is important to consider that LLPS of viroplasms appear only to occur early in the infection, when viroplasm interaction with LDs has not started yet [35,52].

Rotavirus infection leads to the depolymerization of microtubules, which are components of the cytoskeleton; the resulting tubulin granules co-localize with viroplasms [63]. Microtubule depolymerisation can also be achieved by overexpressing NSP2 inside cells [63]. Using chemical inhibition and siRNA-based gene silencing, proteasomal degradation was found to be involved in the formation of viroplasms, and essential for a fully functional viral replication [64,65].

Viroplasms sequester the early steps of viral morphogenesis and viral RNA replication from the cytoplasm, by excluding cellular proteins such as those forming stress granules and P bodies [66].

Interestingly, in RV-infected cells, the expression of miRNA7 targeting the NSP5 gene is upregulated, leading to the inhibition of viroplasm formation and a decrease in the yield of infectious viral progeny production [67]. This effect was reproduced in RV-infected suckling mice, treated with miRNA7 agonists and antagonists [67], further supporting the key role of NSP5 in viroplasm formation.

## 6. Rotavirus Replication Steps Inside Viroplasms

**Figure 4:**
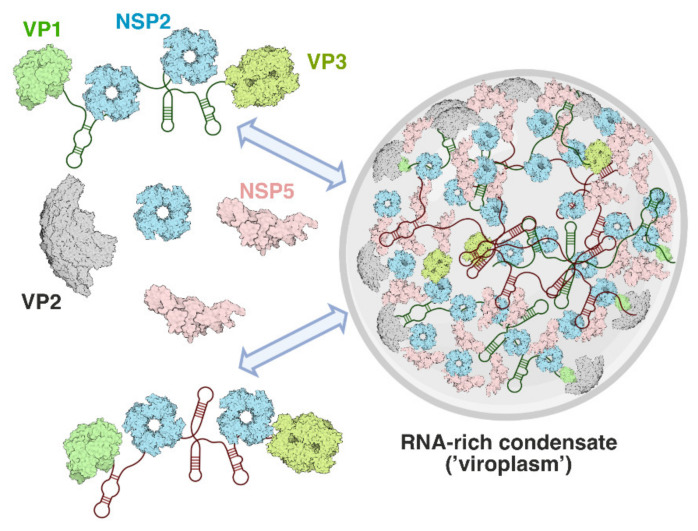
(diagram). Assembly of viroplasms during rotavirus infection. NSP5 (pink) acts as a scaffold that recruits RNA chaperone NSP2 (cyan doughnut-shaped octamers), and other RNA-binding clients (e.g., VP1, VP2 and VP3). NSP5 and NSP2 undergo condensation at low micromolar concentrations, forming protein droplets known as ‘viroplasm-like structures’ [21]. Viral transcripts undergo enrichment in these condensates, likely through protein–RNA interactions between NSP2, or VP1 and the scaffolding protein NSP5. VP1 recognizes 3′terminal sequences of all eleven distinct (+)ssRNA transcripts, thus allowing them to enter the dense NSP5/NSP2 phase of a viroplasm. Other multivalent RNA-binding proteins, i.e., the viral capping enzyme VP3, form the ribonucleoprotein (RNP) complexes that can be absorbed into the NSP2/NSP5 condensates. The unique molecular environment (concentrated viral (+)ssRNA transcripts, RNA chaperone NSP2, the scaffolding protein NSP5, and the inner core protein VP2) are expected to be conducive to the multi-RNA genome assembly and packaging steps. Adapted from Borodavka et al. [15] and Geiger et al. [35].

The earliest identified minimal ‘replication complex’ consists of VP1, VP3 and individual ss(+)RNA pre-genomic segments, in which the RNA forms a putative panhandle structure [68,69,70]. These individual replication complexes are assorted by RNA–RNA interactions in a sequence-specific manner, mediated by NSP2 binding to the ss(+)RNA and acting as an RNA chaperone, which changes RNA folding and leads to the formation of stable inter-segment contacts [15]. Inter-segmental RNA–RNA interactions are believed to control selective and equimolar pre-genome RNA assortment, followed by/concurrent with their co-condensation with 120 copies of VP2 to form the inner core particle [71]. While NSP2 is recognized to be involved in the assortment and packaging of the 11 RNA segments into the core, it has to be evicted from the progeny particle by a still unknown mechanism [15]. This model, which still has knowledge gaps, is diagrammatically shown in Figure 4.

The packaging mechanism of RV RNAs is different from that of the pre-genomic RNA segments of phi6-like bacteriophages (of the *Cystoviridae* family) [72]. As NSP5 also interacts with the RdRp VP1 [73] and VP2 [74], VP1 and VP2 accumulate in viroplasms. Biochemical and structural studies of RV replication revealed that genome replication requires the presence of VP2 [71,75,76,77,78], which also accumulates in viroplasms. The viral replication complexes are located at the 5-fold symmetry positions inside the cores, with each segment being associated with a dedicated complex of enzymes, as shown by individual (+)ssRNA production in the transcriptional mode [79]. Interestingly, there are functional restrictions in the interactions of VP1 and VP2 molecules of different genotypes [80]. Whether these protein incompatibilities affect their distribution within viroplasmic condensates remains to be investigated. Early analysis of RV replication intermediates (RI) differentiated pre-core RIs (containing VP1, VP3, NSP2 and NSP5), core RIs (containing VP1, VP3, VP2, NSP2 and NSP5) and single-shelled (double-layered) RIs (containing VP1, VP3, VP2, VP6, NSP2 and NSP5), and their sequential assembly by the addition of VP2 and VP6 has been proposed [81]. Several of those subviral particles were confirmed, and some novel assembly complexes identified by EM [76,82]. It is still not fully understood at which stage of RI particle assembly the VP1 starts to function as a replicase [76]. With genomic dsRNAs having been synthesized, viral cores undergo further encapsidation by the VP6 present in the viroplasms; this can be also achieved in vitro, as shown by transcapsidation studies [83]. Analysis of the VP6 trimer structure [84] revealed the residues located at the interface with VP2, improving our understanding of their functional significance for DLP assembly and their transcriptional activity [85]. The rotavirus VP2-VP6 DLPs are released from the viroplasms into the cytoplasm for further maturation in viroplasm-associated membranes [9], interacting with NSP4, which was proposed to act as an intracellular DLP receptor [86].

## 7. Concluding Remarks

Plasmid-only-based reverse genetic and CRISPR-Csy4 editing techniques have significantly enriched our understanding of the structure–function relationships of viroplasm components during RV replication. The recent recognition that liquid–liquid phase separation plays a role in viroplasm formation will galvanise studies into many open questions in virus assembly and replication from a new angle. A better understanding of the role of LLPS in RV replication will likely shed light on the interplay between viroplasms and host components (e.g., innate immune sensing, interaction with LDs, microtubules, micro-RNAs), which may also become amenable to novel translational project work.

## Data Availability

Data supporting statements in the text are shown. Additional data can be obtained from G.P. and A.B. upon request.
